# Diversity and antimicrobial potential of culturable heterotrophic bacteria associated with the endemic marine sponge *Arenosclera brasiliensis*

**DOI:** 10.7717/peerj.419

**Published:** 2014-06-17

**Authors:** Cintia P.J. Rua, Amaro E. Trindade-Silva, Luciana R. Appolinario, Tainá M. Venas, Gizele D. Garcia, Lucas S. Carvalho, Alinne Lima, Ricardo Kruger, Renato C. Pereira, Roberto G.S. Berlinck, Rogério A.B. Valle, Cristiane C. Thompson, Fabiano Thompson

**Affiliations:** 1 Instituto de Biologia, Universidade Federal do Rio de Janeiro, Rio de Janeiro, Brazil; 2 Faculdade de Tecnologia e Ciências, Salvador, Bahia, Brazil; 3 Departamento de Biologia Celular, Universidade de Brasília, Brasília, DF, Brazil; 4 Instituto de Biologia, Universidade Federal Fluminense, Niterói, RJ, Brazil; 5 Instituto de Química de São Carlos, Universidade de São Paulo, São Carlos, SP, Brazil; 6 SAGE-COPPE Centro de Gestão Tecnológica, Universidade Federal do Rio de Janeiro, Rio de Janeiro, Brazil

**Keywords:** Symbiont, Antimicrobial activity, Bacteria, Porifera

## Abstract

Marine sponges are the oldest Metazoa, very often presenting a complex microbial consortium. Such is the case of the marine sponge *Arenosclera brasiliensis*, endemic to Rio de Janeiro State, Brazil. In this investigation we characterized the diversity of some of the culturable heterotrophic bacteria living in association with *A. brasiliensis* and determined their antimicrobial activity. The genera *Endozoicomonas* (*N* = 32), *Bacillus* (*N* = 26), *Shewanella* (*N* = 17), *Pseudovibrio* (*N* = 12), and *Ruegeria* (*N* = 8) were dominant among the recovered isolates, corresponding to 97% of all isolates. Approximately one third of the isolates living in association with *A. brasiliensis* produced antibiotics that inhibited the growth of *Bacillus subtilis*, suggesting that bacteria associated with this sponge play a role in its health.

## Introduction

Marine sponges are the oldest Metazoans living on Earth, and are considered to harbor one of the richest microbial symbiont communities among marine invertebrates (Taylor et al., 2007). More than 47 bacterial phyla have already been detected in sponges (Reveillaud et al., 2014), some of which are almost exclusively found in these marine invertebrates (Taylor et al., 2012). For example, a new species of *Shewanella* was isolated from sponges (Lee et al., 2006) and a new candidate phyla, named Poribacteria, was proposed to occur almost exclusively in association with sponges (Fieseler et al., 2004; Taylor et al., 2012). In addition to the high taxonomic diversity of bacteria coexisting in a single sponge individual, experimental evidences show that sponge-associated microbial symbionts produce secondary metabolites (Noyer, Thomas & Becerro, 2011; Sacristán-Soriano et al., 2011; Schneemann et al., 2011; Blunt et al., 2012). Moreover, sponge microbial symbionts present high metabolic diversity, based on variable sources of carbon, nitrogen, oligoelements and other essential nutrients (Hoffmann et al., 2005; Weisz et al., 2007). The investigation of the symbiotic microbial community diversity constitutes an essential aspect to understand marine sponge ecology and possible biotechnological applications.

A particularly relevant example of association between microorganisms and sponges occurs between a *Micromonospora* strain, which produces a bioactive alkaloid, and a haplosclerid sponge (Taylor et al., 2007). The direct assignment of a specific sponge-associated isolate of biotechnological relevance is however a rare occurrence, even considering the increasing efforts towards the discovery of sponge associated bacteria, cyanobacteria and fungi capable of producing biologically active secondary metabolites (Wang, 2006; Öztürk et al., 2013).

Generally, the assignment of an organism as the producer of a given compound is not straightforward, since the production of the compound may be result of the cooperation between symbionts and the host or among the symbionts (Hentschel et al., 2012). Many sponge bioactive compounds are structurally similar to products of prokaryote polyketide synthases (PKS) and non-ribosomal peptide synthases (NRPS). These two enzyme families are involved in many biosynthetic pathways of natural products (Piel, 2009). Setting the bacterial origin of bioactive compounds offers the possibility for heterologous large-scale production (Graça et al., 2013).

The marine sponge *Arenosclera brasiliensis* Muricy & Ribeiro, 1999 (Haplosclerida: Callyspongiidae), is endemic from the upwelling region of Cabo Frio, Rio de Janeiro State, Brazil (Muricy et al., 2011), and the natural source of antibiotic and cytotoxic polycyclic alkaloids (Torres et al., 2000; Berlinck et al., 2004). Evidences suggesting that bacteria associated with *A. brasiliensis* are the source of bioactive compounds of pharmacological interest are numerous. Recent metagenomic studies uncovered the highly diverse microbial community of *A. brasiliensis* (Trindade-Silva et al., 2012). Metagenomic analysis of *A. brasiliensis* also showed a high diversity of bacteria-derived genes encoding for a ketosynthase (KS) domain of the multifunctional type I polyketide synthases (PKS-I) (Trindade-Silva et al., 2013). Such feature is of particular interest, since it has been hypothesized that alkaloids from haplosclerid sponges are assembled from polyketide precursors related to type I PKS enzymology (Fontana, 2006). Moreover, it has been shown that polyketide-derived secondary metabolites isolated from sponges and other marine invertebrates frequently are metabolic products of associated bacteria (Piel et al., 2004; Piel, 2006; Piel, 2009). Consequently, one could hypothesize that the biosynthetic pathway leading to the formation of *A. brasiliensis* alkaloids may be of bacterial origin. Since many bacterial PKS producers are culturable, the isolation and characterization of *A*. *brasiliensis* associated-bacteria is the first step to establish the identity of sponge microorganisms which may have biotechnological potential.

The purpose of the present investigation was the isolation and taxonomic identification of bacteria associated with *A. brasiliensis* and to evaluate the ability of the bacterial isolates to produce antibacterial metabolites.

## Material and Methods

### Sample collection and bacterial isolation

Specimens of *A. brasiliensis* were collected at approximately 10 m depth by Scuba, at João Fernandinho Beach, Búzios, state of Rio de Janeiro (22°44′49″S 41°52′54″W) in January 2011. The sampling site is characterized by tropical conditions with very low annual rainfall. Water temperature ranges from 19 to 27 °C and mean daily air temperatures range from 16 to 28 °C. This part of the coast is influenced by upwelling of cold, nutrient-rich waters and the phenomenon is associated with local wind regime, bathymetry and seasonality (Yoneshigue, 1985; Guimaraens & Coutinho, 1996). Sponge specimens were transported to the laboratory in 20 L containers under controlled temperature (24 °C) in aerated seawater for approximately 3 h. In the laboratory, two specimens were three times washed in 10 mL sterile seawater for removing unassociated micro-organisms. Then, these specimens were dried by gently pressing on sterile paper towels. A piece of sponge (approx. 1 g) was homogenized in 5 mL sterile saline solution (3% NaCl in distilled water). The homogenate was 10-fold serially diluted starting with 100 µL homogenate in 900 µL sterile water to obtain dilutions of 10^−1^; 10^−2^ and 10^−3^ of the initial concentration. A 100 µL aliquot of each dilution was plated onto each of two growth media: BD Difco™ Marine Agar 2216 and MacConkey Agar, both supplemented with Amphotericin B (1 µg/mL) to inhibit the growth of fungi. Isolated bacterial colonies from both media were selected and streaked again at least twice to obtain pure cultures. Pure cultures were preserved in glycerol 20% at −80 °C.

### DNA extraction and 16S rRNA sequencing

Bacterial isolates were grown on the same media used for isolation and incubated for 2–3 days aerobically at 30 °C. Bacterial isolates were harvested from agar plates and suspended in 200 µL of ultrapure sterile water. The bacterial suspensions were subjected to boiling for 10 min and freezing for 3 min to lyse the cells. The lysates were centrifuged for 1 min (4 °C at 13,000 g) for pelleting the cells debris. The resulting supernatant DNA solutions were used for the Polymerase Chain Reaction (PCR) as it follows.

Partial 16S rRNA gene sequences were amplified using the primers 27F (AGA GTT TGA TCM TGG CTC AG) (Lane, 1991) and 1093R (GTT GCG CTC GTT GCG GGA CT) (Thompson et al., 2001). A single PCR contained 25 µL with 1 µL DNA (10–80 ng); 1.5 mM MgCl_2_; 200 µM of each deoxynucleoside triphosphate; 0.5 µM forward and reverse primers and 1 U goTaq DNA polymerase. PCR cycles consisted of initial denaturation at 95 °C for 5 min and cycles of 95 °C for 1.5 min, annealing at 55–51 °C (the temperature was decreased by 1 °C between consecutive steps) for 1 min and the extension at 72 °C for 2 min. The three first cycles containing the higher annealing temperatures (55–53) were repeated 2 times followed by 5 repeats of the cycle with annealing at 52 °C. Finally, 28 repeats of the cycle with annealing at 51 °C and final extension at 72 °C for 4 min. PCR amplicons were checked and the quantity was estimated by electrophoresis on 1% agarose gels and KODAK MI SE—Molecular Imaging Software.

The PCR products were purified using ExoSap-IT (USB Corporation, Cleveland, OH, USA) and sequenced for both the forward and reverse strands using ABI Big Dye chemistry on an ABI 3500 DNA sequencer (Applied Biosystems, Foster City, CA, USA).

### Phylogenetic reconstruction

All sequences were edited using SEQMANII software (DNASTAR, Inc.) and aligned in Clustal X with MEGA5 (Tamura et al., 2011). The aligned sequences were visually inspected and edited when necessary.

Partial 16S rRNA gene sequences were analyzed first via BLASTn tool (nucleotide collection database) (Altschul et al., 1990) and RDP Naive Bayesian rRNA Classifier Version 2.6, Sep 2013 (Wang et al., 2007) to aid selection of most closely related reference sequences. The alignment containing isolates and reference sequences was used first for determining the substitution model of evolution using Modeltest (Posada & Crandall, 1998) for maximum likelihood (ML) and neighbor-joining (NJ) phylogenetic reconstruction using MEGA5. Model-test result was Tamura Nei model with Gamma distributed rates among sites (TrN + G). Both phylogenetic methods were run with 1,000 bootstrap replicates.

Gene sequences obtained in this study are available through the website TAXVIBRIO (http://www.taxvibrio.lncc.br/) and at GenBank database under the accession numbers KJ372433 to KJ372528.

### Antimicrobial activity assays

Antimicrobial activity against *Bacillus subtilis* was assessed using an adapted top agar method (Brady, 2007). Briefly, bacterial isolates were grown at 28 °C in the center of squares (5.76 cm^2^) marked in 245 mm × 245 mm sterile plates (Corning) during 6 days. After this period, the plates containing isolates were exposed to 3 mL of chloroform, during 1 h in order to kill the isolates, and then the plates were left opened to ensure complete chloroform evaporation. An overlay with *B. subtilis* LMG 7135^*T*^ was poured over the killed isolates. The soft agar medium containing *B. subtilis* was prepared as follows: firstly, *B. subtilis* was grown in 50 mL Luria Broth (LB) medium at 28 °C for 16 h. Then, 200 mL of LB soft agar (0.7% agar) at 45 °C was added to *B. subtilis* inoculum. This semi solid medium containing *B. subtilis* was then poured over those killed isolates. The antimicrobial activity was taken into account with the appearance of haloes in *B. subtilis* confluent growth after 24 and 48 h. Four replicates were performed for each isolate in order to confirm antimicrobial activity. Control tests corresponded to squares where no isolate was inoculated and complete growth of *B. subtilis* was expected.

Antimicrobial activity against *Vibrio sinaloensis* LMG 25238^*T*^ was also screened by a modified double-layer method previously described (Westerdahl et al., 1991). Briefly, the isolates were individually cultivated in Trypic Soil Agar (TSA) (NaCl 3%) for 48 h at 30 °C, under aerobic conditions. Dilutions on sterile distilled water (NaCl 3.0%) were adjusted using a spectrophotometer to optical density of 0.08–0.1 at 625 nm, corresponding to approx. 10^8^ cells mL^−1^ and seeded, using a steers replicator, on plates containing marine agar. After incubation for 48 h at 30 °C, under aerobic conditions, growing spots were observed. *V. sinaloensis* (10^8^ cels mL^−1^) was incorporated into sterile fluid semisolid marine medium (0.5% agar) and spread over the plates containing the isolated bacterial spots. Plates containing the isolates and the test-strain were incubated for 48 h at 30 °C. The bacterial growth inhibition was observed by determining the presence of the inhibition zones. The assay was performed in triplicate.

## Results

**Figure 1:**
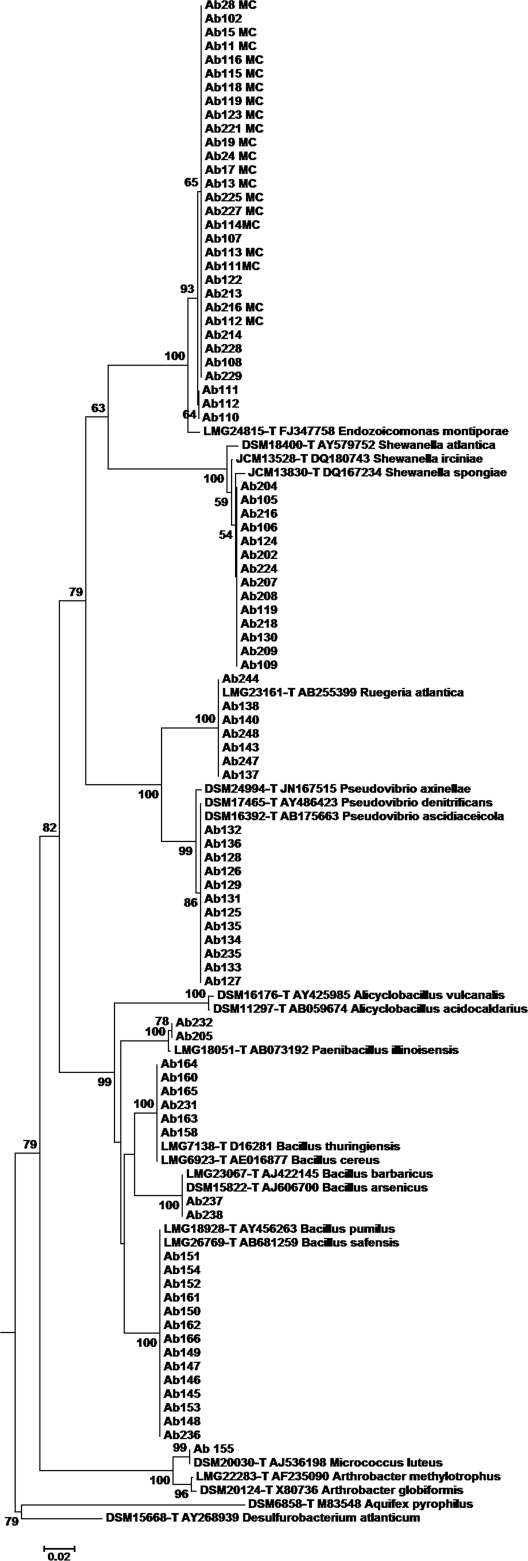
Phylogenetic tree of partial 16S rRNA sequences (302 bp) of *A. brasiliensis* isolates and type strains sequences. The phylogeny reconstruction was based on neighbor-joining method and 1,000 bootstrap replications.

A total of 98 culturable bacterial isolates were obtained and their partial 16S rRNA gene sequences were compared with the GenBank and RDP databases in order to find the closest neighbors and the respective type-strain. Genera *Endozoicomonas* (*N* = 32), *Bacillus* (*N* = 26), *Shewanella* (*N* = 17), *Pseudovibrio* (*N* = 12), and *Ruegeria* (*N* = 8) were dominant among the culturable microbiome of *A. brasiliensis*, corresponding to approx. 97% of all isolates (Complete list of isolates is on http://www.taxvibrio.lncc.br/). The three remaining isolates were closely related to *Paenibacillus* (*N* = 2) and *Micrococcus* (*N* = 1). Besides the taxonomic identification through comparisons of 16S rRNA sequences with databases, the 16S rRNA sequences were grouped using phylogenetic methods. The *Endozoicomonas* isolates presented sequence with 97.5% similarity towards the type strain of *E. montiporae* (Fig. 1; Fig. S1). The *Shewanella* isolates clustered all together, having at maximum 98% 16S rRNA sequence similarity towards *S. irciniae* type strain (Fig. 1; Fig. S2). *Ruegeria* isolates had 100% similarity with *Ruegeria atlantica* type strain (Fig. 1; Fig. S3). Part of *Pseudovibrio* isolates had 100% similarity with the sequence of *P. denitrificans* type strain and the remainder had identical 16S rRNA sequences with the *P. asceidicola* type strain (Fig. 1; Fig. S4). *Paenibacillus* isolates showed 99.8% similarity with *P. illinoiensis* type strain. *Bacillus* isolates had 100% similarity with the *B. pumillus*, *B. safensis*, *B. thuringiensis*, *B*. *cereus*, *B. barbaricus* and *B. arsenicus* type-strains (Fig. 1; Figs. S5–S6).

### Antimicrobial activity

Antimicrobial activity was detected in 28.5% of the *A. brasiliensis* bacterial isolates of different species (Table 1). *Bacillus subtilis* test strain was susceptible to the supernatant of different *A. brasiliensis* bacterial isolates. The antimicrobial activity was widespread through different taxonomic groups of bacteria. *Vibrio sinaloensis* was susceptible to four isolates: two isolates closely related to *Shewanella spongiae* (i.e. Ab 105 and Ab 216), and two isolates closely related to *Pseudovibrio ascidiaceicola* (i.e. Ab 133 and Ab134).

**Table 1:** Bacterial isolates of *A. brasiliensis* showed antimicrobial activity. Results of antimicrobial activity tests. Isolates belonging to different species inhibit the growth of *B. subtilis*.

*B. subtilis* was susceptible to the following isolates	
Genus	Isolates
*Endozoicomonas montiporae*	Ab107/Ab227MC/Ab112/Ab213
*Shewanella irciniae*	Ab114/Ab 101/Ab105/Ab109/Ab202/Ab216
*Ruegeria atlantica*	Ab137/Ab138
*Pseudovibrio ascidiaceicola*	Ab126/Ab132
*Pseudovibrio denitrificans*	Ab127/Ab133/Ab134
*Paenibacillus illinoiensis*	Ab232
*Bacillus pumillus*	Ab 147/Ab148/Ab153/Ab161/ Ab166
*Bacillus cereus*	Ab158/Ab160/Ab164/Ab233
*Bacillus nanhaiensis*	Ab238

## Discussion

As primitive Metazoans, marine sponges have established long-term associations with an array of microorganisms, which mainly include bacteria and cyanobacteria (Taylor et al., 2007). In the present investigation, we observed that all bacterial genera found among the culturable heterotrophic community of *A. brasiliensis* have already been isolated from other marine sponges, but, the present combination of bacterial genera was never reported within a single marine sponge. Comparisons between culturable bacterial communities from marine sponges are difficult to address since differences in media and culture conditions can significantly influence the isolation and cultivability of distinct bacterial isolates (Sipkema et al., 2011). In spite of this, it seems that some genera are commonly found among marine sponges, such as *Pseudovibrio* (Enticknap et al., 2006; Mohamed et al., 2008; Menezes et al., 2010; Santos et al., 2010; Flemer et al., 2011; Bruck, Reed & McCarthy, 2012; Margassery et al., 2012), *Bacillus* (Hentschel et al., 2001; Webster et al., 2001; Pabel et al., 2003; Lafi, Garson & Fuerst, 2005; Zhu, Li & Wang, 2008; Bruck et al., 2010; Devi et al., 2010; Santos et al., 2010; Flemer et al., 2011; Phelan et al., 2011; Bruck, Reed & McCarthy, 2012; Margassery et al., 2012), and *Ruegeria* (Mohamed et al., 2008; Menezes et al., 2010; Bruck, Reed & McCarthy, 2012; Margassery et al., 2012; Esteves et al., 2013; Haber & Ilan, 2013). Such bacterial genera can be found in distinct marine sponge species collected at distant geographic areas and with low phylogenetic relationship. On the other hand, *Endozoicomonas*, usually referred to as *Spongiobacter* (Mohamed et al., 2008; Flemer et al., 2011), and *Shewanella* (Flemer et al., 2011; Margassery et al., 2012; Haber & Ilan, 2013) are bacterial genera less frequently found within marine sponges (Table S1).

*Endozoicomonas* isolates obtained in this investigation were closely related to those isolates from Australian sponges and corals. Similarly, *Shewanella*, *Ruegeria*, *Pseudovibrio*, *Paenibacillus*, *Bacillus* and *Micrococcus* isolates grouped with related isolates obtained from different sponge species collected at different geographic locations (Figs. S1–S7).

### The diversity and abundance of the genera detected through culture-dependent and culture-independent means have limited overlap

The diversity of culturable bacterial isolates recovered from *A. brasiliensis* presented similarity to the *A. brasiliensis* metagenomes previously analyzed by us (Trindade-Silva et al., 2012). For example, *Pseudovibrio* (*N* = 5), *Ruegeria* (*N* = 23), *Shewanella* (*N* = 16), *Paenibacillus* (*N* = 1) and *Bacillus* (*N* = 1) were observed in the metagenomic sequences analyzed previously, whereas *Endozoicomonas* and *Micrococcus* were not detected in the metagenomes of *A. brasiliensis*. In addition, we identified *Pseudovibrio*, *Ruegeria*, *Shewanella*, *Paenibacillus*, *Bacillus*, and *Micrococcus* in sequence libraries of polyketide synthase (PKS) genes originated from *A. brasiliensis* (Trindade-Silva et al., 2013), indicating some overlap between the culture-dependent and culture-independent studies. It demonstrates that suitable culture conditions can recover some of the bacterial genera detected by metagenomic analysis. Comparisons between culture-dependent and culture-independent approaches suggest that novel culturing approaches are needed in order to cultivate the missing bacterial diversity (Lavy et al., 2014).

### The culturable heterotrophic bacteria of *A. brasiliensis* comprise well known antibiotic producers

Bacterial isolates belonging to the genera *Endozoicomonas*, *Pseudovibrio* and *Paenibacillus* are known for their ability to produce antibiotics. *Pseudovibrio* isolates originated from marine invertebrates such as tunicates (Sertan-de Guzman et al., 2007; Riesenfeld, Murray & Baker, 2008), corals (Rypien, Ward & Azam, 2010) and sponges showed antimicrobial activity (Santos et al., 2010; O’ Halloran et al., 2011). *Pseudovibrio* isolates from corals have antagonist effect against *V. coralliilyticus* (Rypien, Ward & Azam, 2010). In our study, we demonstrate that *Pseudovibrio* isolates of *A. brasiliensis* showed antimicrobial activity against *Bacillus subtilis* and *Vibrio sinaloensis*.

Bourne et al. (2008) found a correlation between the presence of *Endozoicomonas* (= *Spongiobacter*) and the health of corals. The authors argue that *Endozoicomonas* may be excluding potential pathogenic microorganisms from the holobiont. Mohamed and coworkers (2008) showed that an *Endozoicomonas*-related isolate from sponges produce a repertoire of N-acyl homoserine lactones. These quorum-sensing signaling metabolites may help delineate and structure the microbial communities in sponges. In the present study, several *Endozoicomonas* isolates showed antimicrobial activity against *B. subtilis* (Table 1). *Endozoicomonas* may play an ecological role in marine holobionts, shaping the associated microbial community through antimicrobial substances and signaling molecules.

*Paenibacillus* isolates belong to the microbial community of sponges, although they are found less abundantly than *Bacillus* (Phelan et al., 2011). The physiological role of sporeformers within sponge tissues is still unknown (Phelan et al., 2011), but probiotic activity was verified in *Bacillus cereus* and *Paenibacillus* sp. against *Vibrio* spp., conferring high levels of survival of shrimp larvae (Ravi et al., 2007). Additionally, antimicrobial activity was already detected in extracts of *Paenibacillus* isolates (Romanenko et al., 2013).

*Micrococcus* was the only Actinobacteria represented in the culturable community of *A. brasiliensis*, with a single isolate. Actinobacteria is a bacterial phylum commonly observed in invertebrate-associated microbial communities. Moreover, Actinobacteria comprises some of the most ingenious producers of potent antibiotics (Sun et al., 2010). Previous studies showed more diverse and abundant Actinobacteria communities in marine invertebrates than we recovered from *A. brasiliensis*. However, specific Actinobacteria growth conditions and nutrient requirements were not applied in the present study, which are normally required for enriching the isolation of representative isolates of this taxonomic group (Jiang et al., 2008; Sun et al., 2010). The diversity of actinomycetes recovered from *A. brasiliensis* is possibly underestimated; however, the microbes associated with *A. brasiliensis* may be providing protection against pathogens and keeping a healthy interaction with the symbiont community.

## Conclusions

The sponge *A. brasiliensis* yielded a diverse culturable microbial community, including *Endozoicomonas*, *Shewanella*, *Ruegeria*, *Pseudovibrio*, *Bacillus*, and *Paenibacillus* isolates with high potential for antibiotic production. The compounds with antibiotic activity that can be explored as sources of natural products can also be those delineating the taxonomic composition of the associated microbial community. Genomic and post-genomic features of the antibiotic bacterial producers will enable to unveil the genes involved in the antibiotic production and define the chemical nature of the antibiotics. In addition, future studies will investigate whether antibiotics produced by microbes associated with *A. brasiliensis* may serve as chemical defenses in order to control the diversity and abundance of potential environmental pathogens.

## Supplemental Information

10.7717/peerj.419/supp-1 Figure S1 Phylogenetic tree of partial 16S rRNA sequences of *Endozoicomonas* isolates Phylogenetic tree of partial 16S rRNA sequences of *Endozoicomonas* isolates, type strains sequences and database sequences of bacterial strains isolated from marine invertebrates. The numbers of sites used in the phylogenetic reconstructions were 202.

10.7717/peerj.419/supp-2 Figure S2 Phylogenetic tree of partial 16S rRNA sequences of *Shewanella* isolates Phylogenetic tree of partial 16S rRNA sequences of *Shewanella* isolates, type strains sequences and database sequences of bacterial strains isolated from marine invertebrates. The numbers of sites used in the phylogenetic reconstructions were 404.

10.7717/peerj.419/supp-3 Figure S3 Phylogenetic tree of partial 16S rRNA sequences of *Ruegeria* isolates Phylogenetic tree of partial 16S rRNA sequences of *Ruegeria* isolates, type strains sequences and database sequences of bacterial strains isolated from marine invertebrates. The numbers of sites used in the phylogenetic reconstructions were 284.

10.7717/peerj.419/supp-4 Figure S4 Phylogenetic tree of partial 16S rRNA sequences of *Pseudovibrio* isolates Phylogenetic tree of partial 16S rRNA sequences of *Pseudovibrio* isolates, type strains sequences and database sequences of bacterial strains isolated from marine invertebrates. The numbers of sites used in the phylogenetic reconstructions were 365.

10.7717/peerj.419/supp-5 Figure S5 Phylogenetic tree of partial 16S rRNA sequences of *Paenibacillus* isolates Phylogenetic tree of partial 16S rRNA sequences of *Paenibacillus* isolates, type strains sequences and database sequences of bacterial strains isolated from marine invertebrates. The numbers of sites used in the phylogenetic reconstructions were 533.

10.7717/peerj.419/supp-6 Figure S6 Phylogenetic tree of partial 16S rRNA sequences of *Bacillus* isolates Phylogenetic tree of partial 16S rRNA sequences of *Bacillus* isolates, type strains sequences and database sequences of bacterial strains isolated from marine invertebrates. The numbers of sites used in the phylogenetic reconstructions were 407.

10.7717/peerj.419/supp-7 Figure S7 Phylogenetic tree of partial 16S rRNA sequences of *Micrococcus* isolates Phylogenetic tree of partial 16S rRNA sequences of *Micrococcus* isolates, type strains sequences and database sequences of bacterial strains isolated from marine invertebrates. The numbers of sites used in the phylogenetic reconstructions were 427.

10.7717/peerj.419/supp-8 Table S1 Genera of bacteria found in *A. brasiliensis* and other sponge hosts Genera of bacteria found in *A. brasiliensis.* Other sponge hosts where these genera were also found are presented with their references and locality of sample collection.

10.7717/peerj.419/supp-9 Supplemental Information 9 Isolates sequences Raw data

## Additional Information and Declarations

### DNA Deposition

The following information was supplied regarding the deposition of DNA sequences: GenBank accession numbers: KJ372433 to KJ372528.
