# Supplementary figures and images for: Diversity and antimicrobial potential of culturable heterotrophic bacteria associated with the endemic marine sponge Arenosclera brasiliensis
Source: PeerJ. 2014 Jun 17;2:e419. doi: 10.7717/peerj.419 (PMC4081303; doi:10.7717/peerj.419)

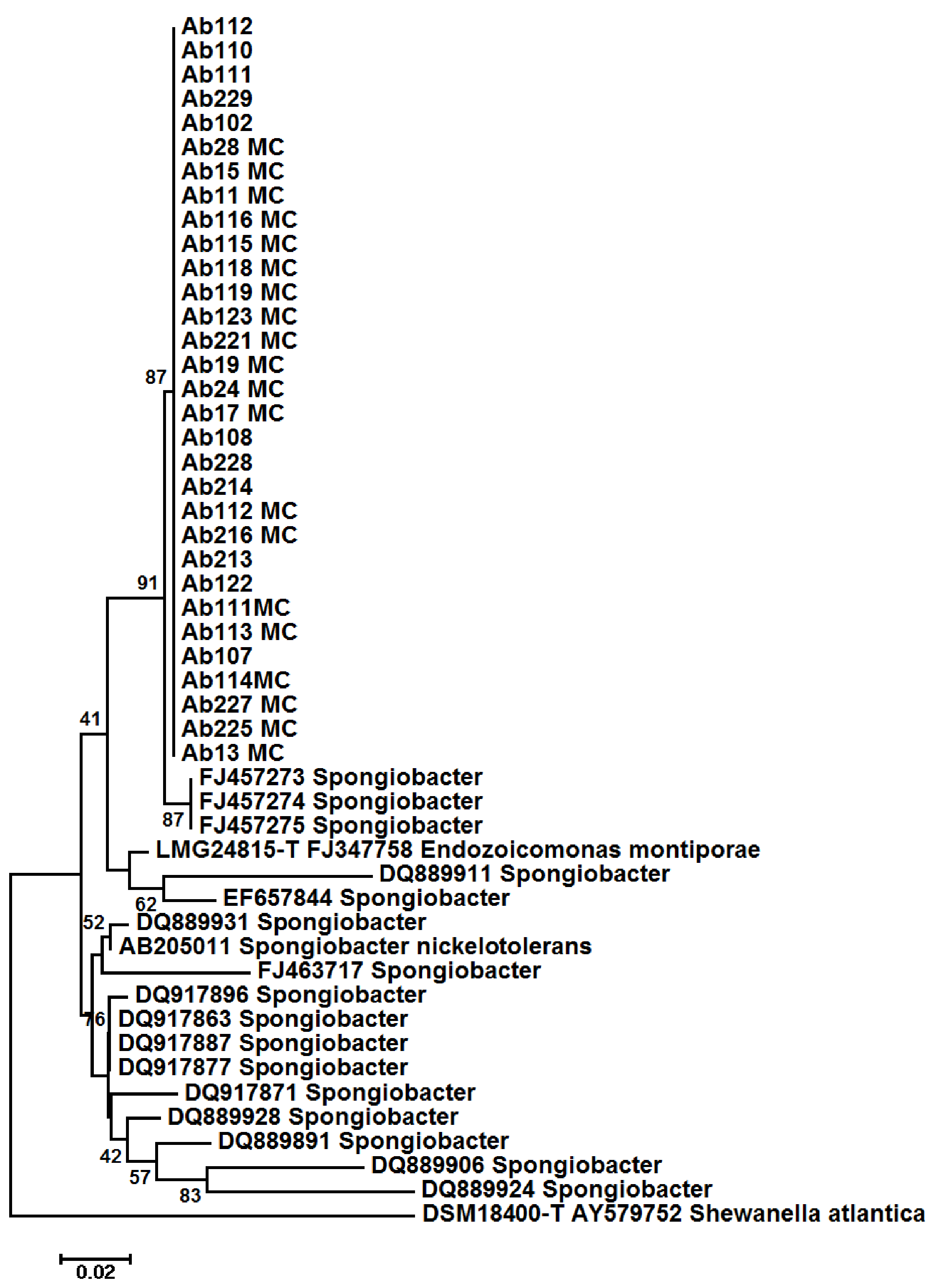

Supplement: Figure S1 — Phylogenetic tree of partial 16S rRNA sequences of Endozoicomonas isolates, type strains sequences and database sequences of bacterial strains isolated from marine invertebrates. The numbers of sites used in the phylogenetic reconstructions were 202. [file peerj-02-419-s001.png]

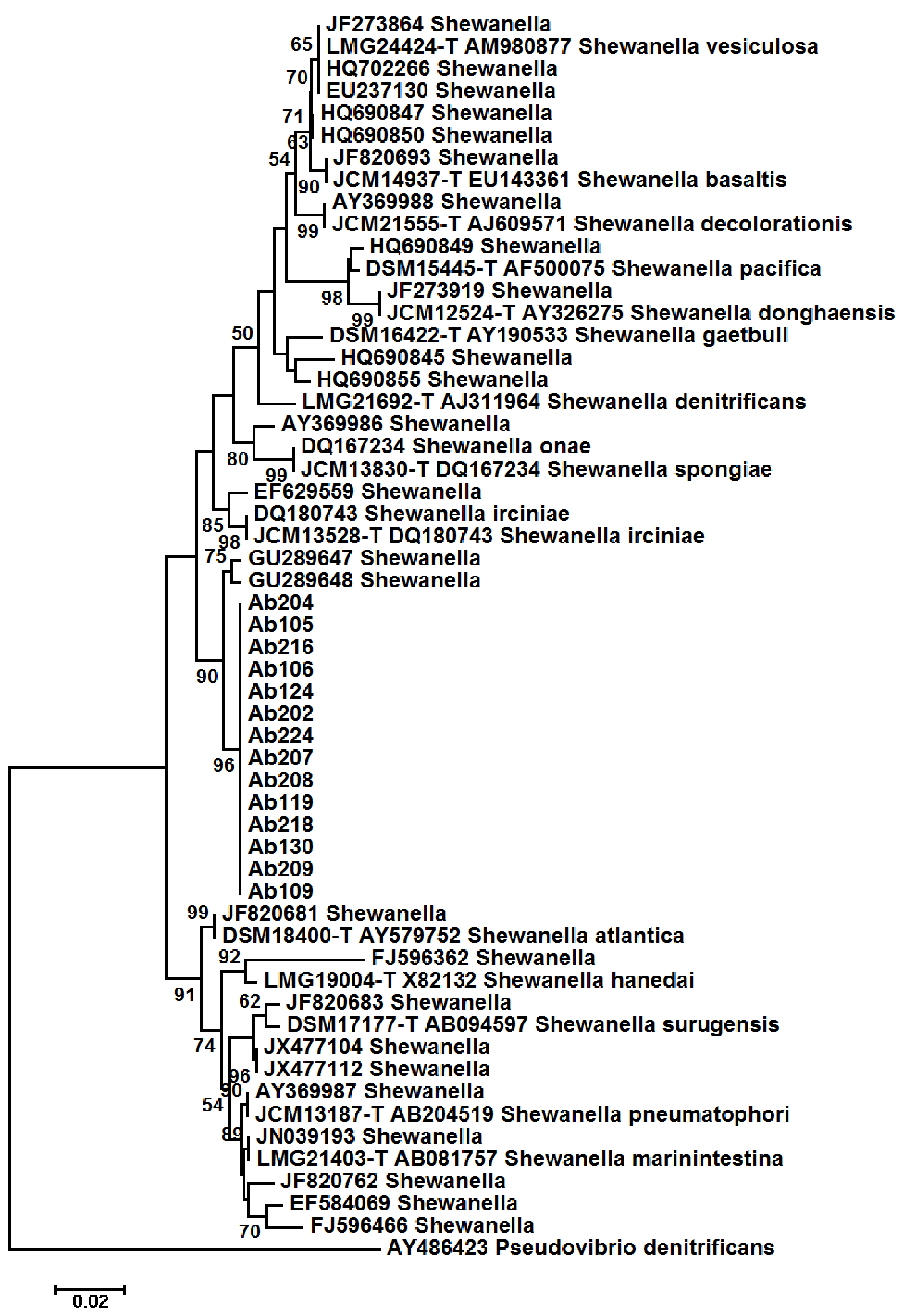

Supplement: Figure S2 — Phylogenetic tree of partial 16S rRNA sequences of Shewanella isolates, type strains sequences and database sequences of bacterial strains isolated from marine invertebrates. The numbers of sites used in the phylogenetic reconstructions were 404. [file peerj-02-419-s002.png]

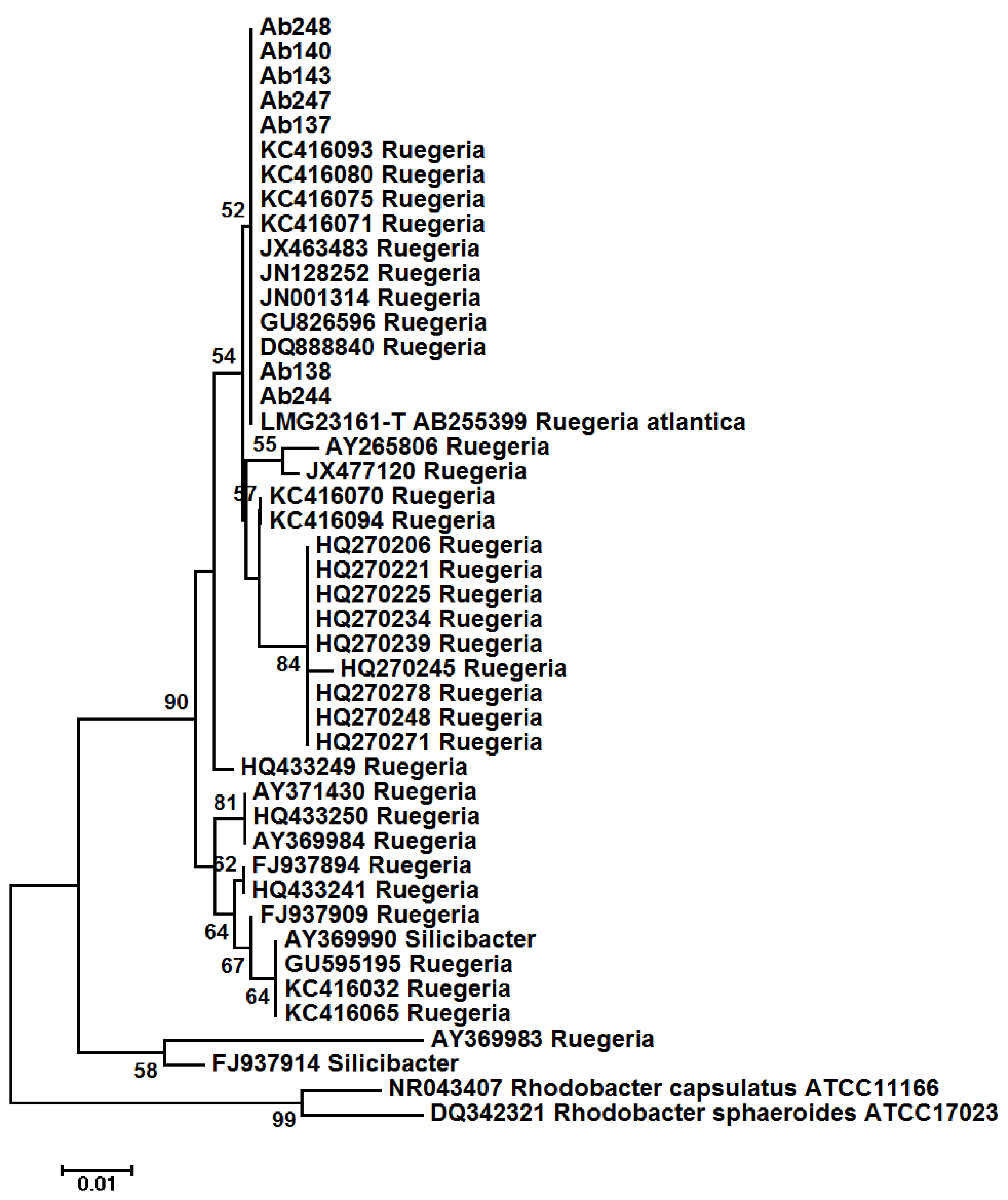

Supplement: Figure S3 — Phylogenetic tree of partial 16S rRNA sequences of Ruegeria isolates, type strains sequences and database sequences of bacterial strains isolated from marine invertebrates. The numbers of sites used in the phylogenetic reconstructions were 284. [file peerj-02-419-s003.png]

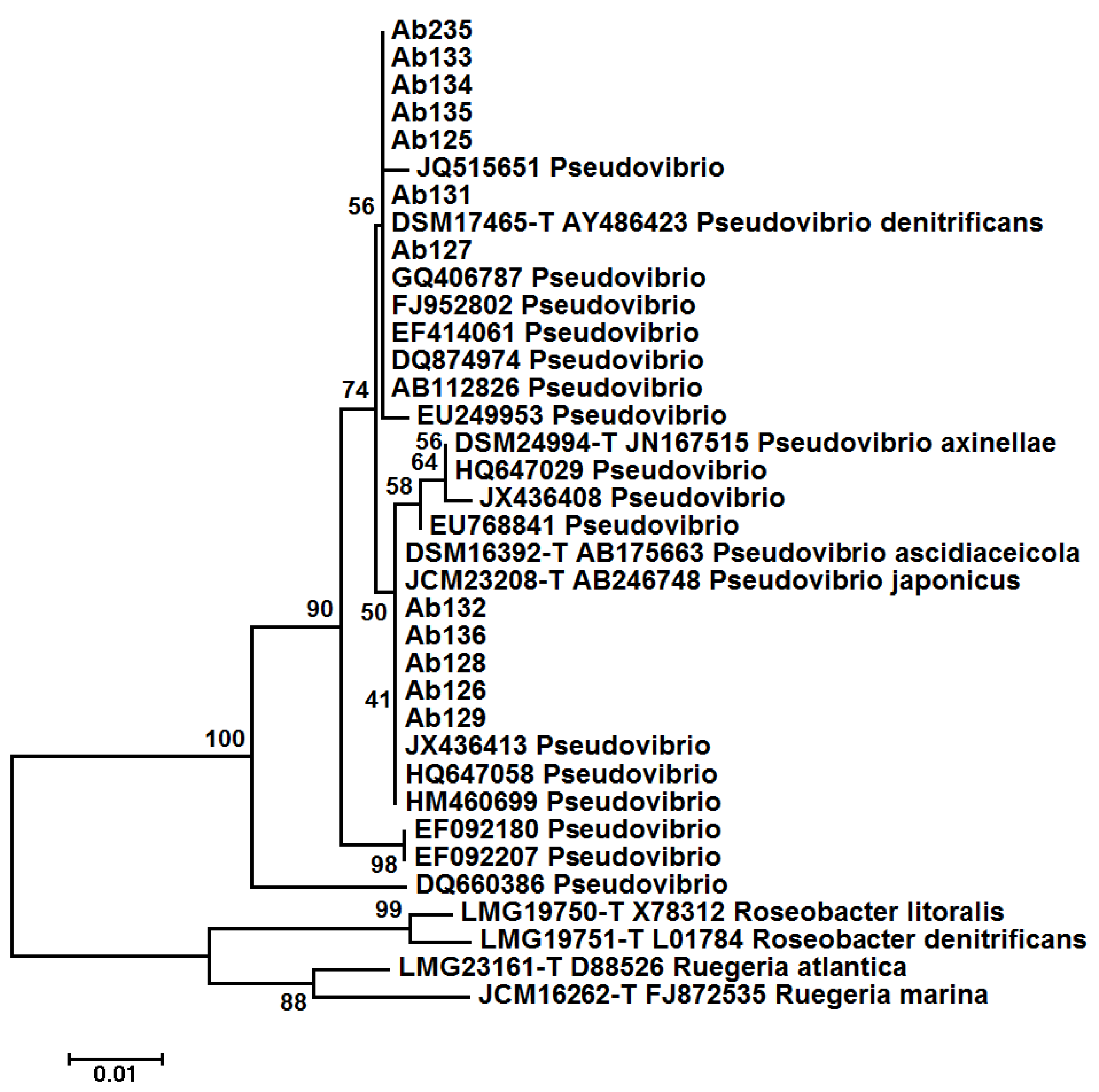

Supplement: Figure S4 — Phylogenetic tree of partial 16S rRNA sequences of Pseudovibrio isolates, type strains sequences and database sequences of bacterial strains isolated from marine invertebrates. The numbers of sites used in the phylogenetic reconstructions were 365. [file peerj-02-419-s004.png]

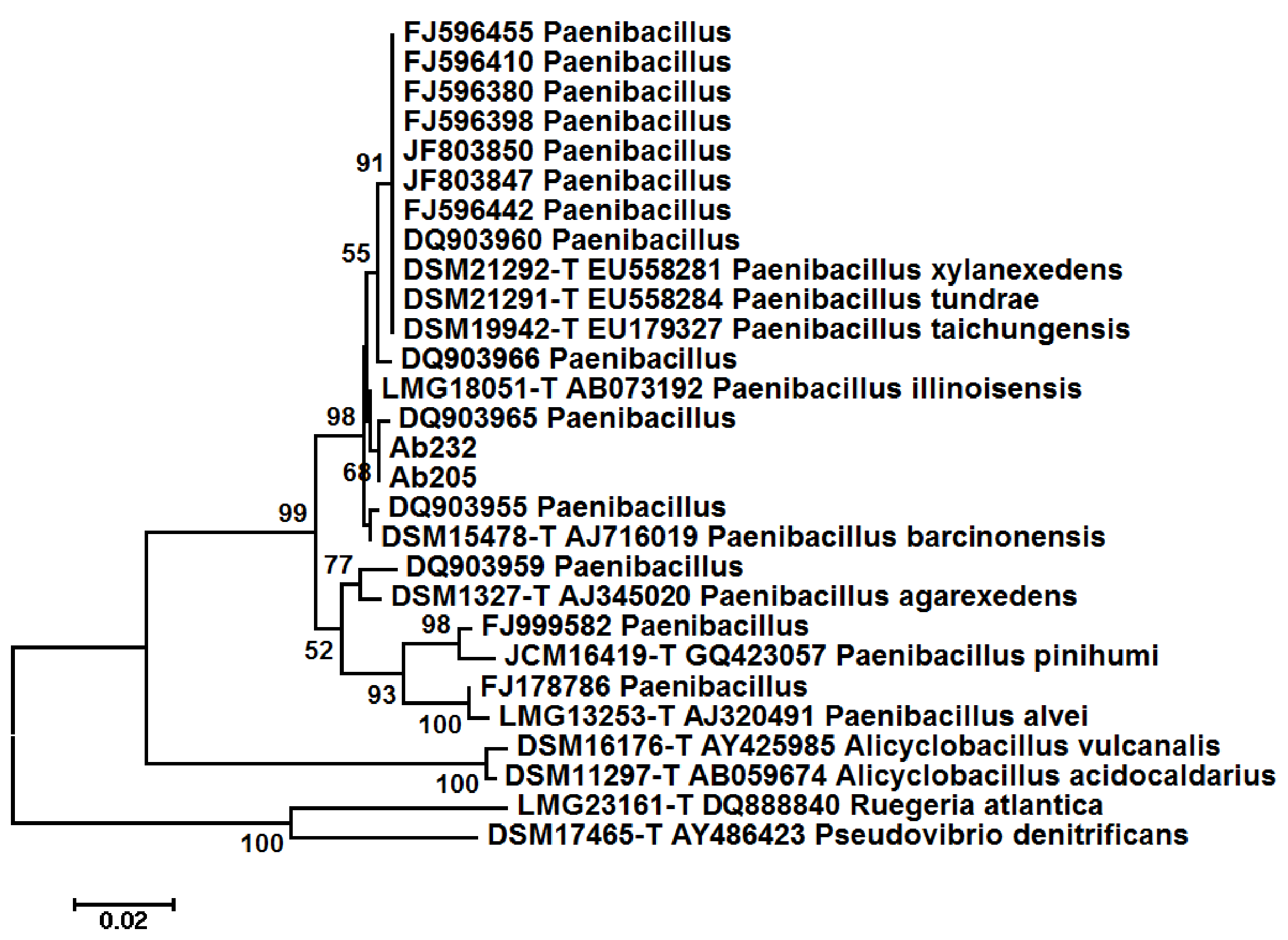

Supplement: Figure S5 — Phylogenetic tree of partial 16S rRNA sequences of Paenibacillus isolates, type strains sequences and database sequences of bacterial strains isolated from marine invertebrates. The numbers of sites used in the phylogenetic reconstructions were 533. [file peerj-02-419-s005.png]

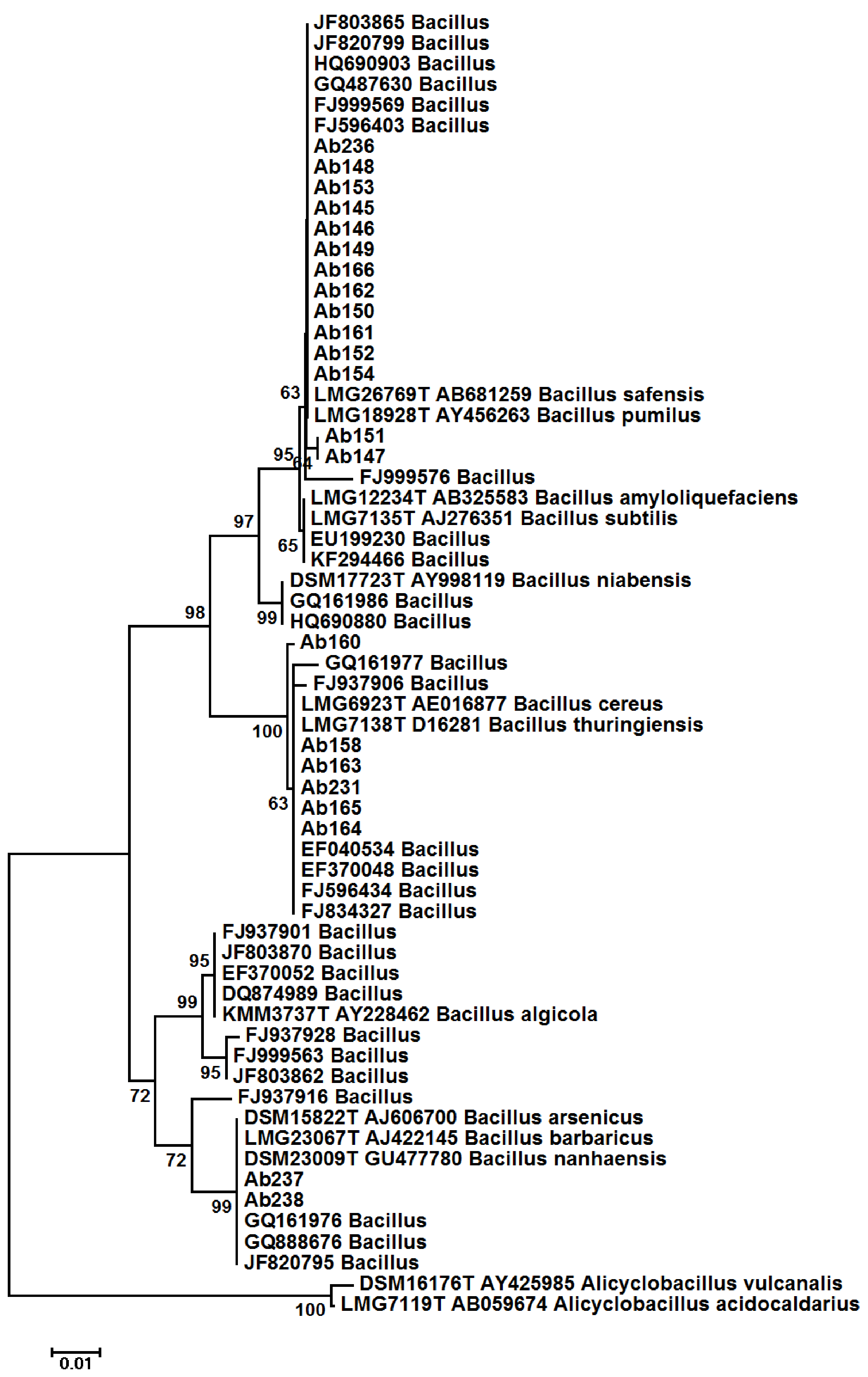

Supplement: Figure S6 — Phylogenetic tree of partial 16S rRNA sequences of Bacillus isolates, type strains sequences and database sequences of bacterial strains isolated from marine invertebrates. The numbers of sites used in the phylogenetic reconstructions were 407. [file peerj-02-419-s006.png]

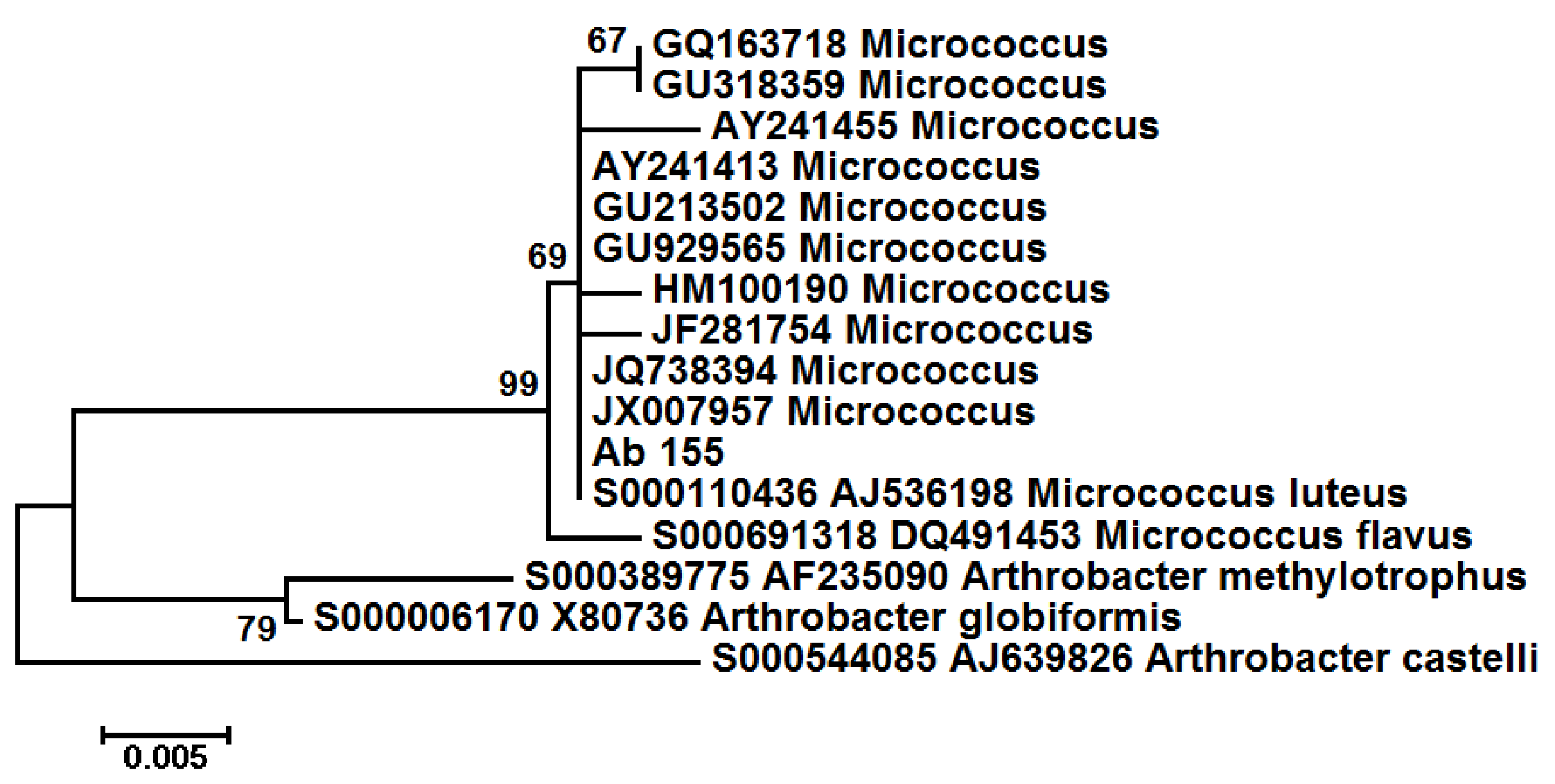

Supplement: Figure S7 — Phylogenetic tree of partial 16S rRNA sequences of Micrococcus isolates, type strains sequences and database sequences of bacterial strains isolated from marine invertebrates. The numbers of sites used in the phylogenetic reconstructions were 427. [file peerj-02-419-s007.png]
