# Supplementary material for: Diversity and antimicrobial potential of culturable heterotrophic bacteria associated with the endemic marine sponge Arenosclera brasiliensis
Source: PeerJ. 2014 Jun 17;2:e419. doi: 10.7717/peerj.419 (PMC4081303; doi:10.7717/peerj.419)
Supplement: Table S1 — Genera of bacteria found in A. brasiliensis. Other sponge hosts where these genera were also found are presented with their references and locality of sample collection. [file peerj-02-419-s008.docx]

| Bacteria genera | **Found in marine sponge of species:** | **Sponge Orders** | **References** | **Locality of sponge collection** |
| --- | --- | --- | --- | --- |
| *Spongiobacter* | *Ircinia strobilina* | Dictyoceratida | Mohamed et al 2008 | Conch Reef, Key Largo, Florida |
|  | *Suberites carnosus* | Hadromerida | Flemer et al 2011 | Cork - Ireland |
|  | Sarcotragus spinosulus | Dictyoceratida | Esteves et al 2013 | Portugal |
|  | *Axinella* spp. | Halichondrida | Haber & Ilan 2013 | Mediterranean - Israel |
| *Shewanella* | *Suberites carnosus* | Hadromerida | Flemer et al 2011 | Cork - Ireland |
|  | *Eurypon major* | Poecilosclerida | Margassery et al 2012 |  |
|  | *Ircinia variabilis* | Dictyoceratida | Esteves et al 2013 | Portugal |
|  | Sarcotragus spinosulus |  |  |  |
|  | *Axinella* spp. | Halichondrida | Haber & Ilan 2013 | Mediterranean - Israel |
| *Ruegeria* | *Discodermia dissoluta* | Lithistida | Bruck et 2012 | Bahamas |
|  | *Mycale laxissima* | Poecilosclerida | Mohamed et al 2008 | Conch Reef, Key Largo, Florida |
|  | *Amphilectus fucorum* |  | Margassery et al 2012 | Cork - Irlanda |
|  | *Eurypon major* |  |  |  |
|  | *Eurypon major* |  |  |  |
|  | *Amphimedon viridis* | Haplosclerida | Menezes et al 2010 | São Paulo - Brazil |
|  | *Dragmacidon reticulata* | Halichondrida |  |  |
|  | *Ircinia variabilis* | Dictyoceratida | Esteves et al 2013 | Portugal |
|  | *Sarcotragus spinosulus* |  |  |  |
|  | *Axinella* spp. | Halichondrida | Haber & Ilan 2013 | Mediterranean - Israel |

| Bacteria genera | **Found in marine sponge of species:** | **Sponge Orders** | **References** | **Locality of sponge collection** |
| --- | --- | --- | --- | --- |
| *Pseudovibrio* | *Mycale laxissima* | Poecilosclerida | Mohamed et al 2008 | Conch Reef, Key Largo, Florida |
|  | *Mycale microsigmatosa* |  | Santos et al 2010 | Brazil |
|  | *Mycale laxissima* |  | Enticknap et al 2006 | Key Largo, Florida |
|  | *Clathria prolifera* |  |  | Chesapeake Bay, USA |
|  | *Monanchora unguifera* |  |  | Jamaica |
|  | *Amphilectus fucorum* |  | Margassery et al 2012 | Cork - Ireland |
|  | *Eurypon major* |  |  |  |
|  | *Acanthostrongylophora sp.* | Haplosclerida | Enticknap et al 2006 | Sulawesi, Indonesia |
|  | *Niphates digitales* |  |  | Key Largo, Florida |
|  | *Didiscus oxeata* | Halichondrida |  | Jamaica |
|  | *Axinella corrugata* |  |  | Key Largo, Florida |
|  | *Discodermia dissoluta* | Lithistida | Bruck et 2012 | Bahamas |
|  | *Suberites carnosus* | Hadromerida | Flemer et al 2011 | Cork - Ireland |
|  | *Paraleucilla magna* | Leucosolenida | Santos et al 2010 | Brazil |
|  | *Clathrina aurea* | Clathrinida |  |  |
|  | *Amphimedon viridis* | Haplosclerida | Menezes et al 2010 | São Paulo - Brazil |
|  | *Mycale laxissima* | Poecilosclerida |  |  |
|  | *Ircinia variabilis* | Dictyoceratida | Esteves et al 2013 | Portugal |
|  | *Sarcotragus spinosulus* |  |  |  |
|  | *Axinella* spp. | Halichondrida | Haber & Ilan 2013 | Mediterranean - Israel |
| *Paenibacillus* | *Suberites zeteki* | Hadromerida | Zhu et al 2008 | Hawaii |
|  | *Haliclona simulans* | Haplosclerida | Phelan et al 2011 | West coast of Ireland |
|  | *Geodia spp.* | Astrophorida | Bruck et 2010 | Straits of Florida |

| Bacteria genera | **Found in marine sponge of species:** | **Sponge Orders** | **References** | **Locality of sponge collection** |
| --- | --- | --- | --- | --- |
| *Bacillus* | *Suberites zeteki* | Hadromerida | Zhu et al 2008 | Hawaii |
|  | *Leucosolenia sp.* | Leucosolenida | Flemer et al 2011 | Cork - Irlanda |
|  | *Rhopaloeides odorabile* | Dictyoceratida | Webster et al 2001 | Great Barrier Reef, Australia |
|  | *Aplysina aerophoba* | Verongida | Pabel et al 2003 | Banyuls-sur-Mer - France |
|  | *Haliclona simulans* | Haplosclerida | Phelan et al 2011 | West coast of Ireland |
|  | *Amphilectus fucorum* | Poecilosclerida | Margassery et al 2012 | Cork - Irlanda |
|  | *Eurypon major* |  |  | Cork - Irlanda |
|  | *Geodia spp.* | Astrophorida | Bruck et al 2010 | Straits of Florida |
|  | *Discodermia dissoluta* | Lithistida | Bruck et al 2012 | Bahamas |
|  | *Petromica citrina* |  | Santos et al 2010 | Brazil |
|  | *Haliclona sp.* | Haplosclerida |  |  |
|  | *Dragmacidon reticulatus* | Halichondrida |  |  |
|  | *Halichondria sp.* |  | Devi et al 2010 | West Coast of India |
|  | *Aplysina aerophoba / cavernicola* | Verongida | Hentschel et al 2001 | Mediterranean - France |
|  | *Pseudoceratina clavata* |  | Lafi et al 2005 | Great Barrier Reef, Australia |
|  | *Rhabdastrella globostellata* | Astrophorida |  |  |
|  | *Mycale laxissima* | Poecilosclerida | Menezes et al 2010 | São Paulo - Brazil |
|  | *Dragmacidon reticulata* | Halichondrida |  |  |
|  | *Geodia corticostylifera* | Astrophorida |  |  |
|  | *Axinella* spp. | Halichondrida | Haber & Ilan 2013 | Mediterranean - Israel |
| *Micrococcus sp.* | *Discodermia dissoluta* | Lithistida | Bruck et al 2012 | Bahamas |
|  | *Mycale laxissima* | Poecilosclerida | Menezes et al 2010 | São Paulo - Brazil |
|  | *Dragmacidon reticulata* | Halichondrida |  |  |
|  | *Geodia corticostylifera* | Astrophorida |  |  |
|  | Sarcotragus spinosulus | Dictyoceratida | Esteves et al 2013 | Portugal |
|  | *Axinella* spp. | Halichondrida | Haber & Ilan 2013 | Mediterranean - Israel |
